# Targeting of Basophil and Mast Cell Pro-Allergic Reactivity Using Functionalised Gold Nanoparticles

**DOI:** 10.3389/fphar.2019.00333

**Published:** 2019-03-29

**Authors:** Inna M. Yasinska, Luigi Calzolai, Ulrike Raap, Rohanah Hussain, Giuliano Siligardi, Vadim V. Sumbayev, Bernhard F. Gibbs

**Affiliations:** ^1^Medway School of Pharmacy, Universities of Kent and Greenwich, Chatham Maritime, United Kingdom; ^2^European Commission, Joint Research Centre, Ispra, Italy; ^3^Division of Experimental Allergology and Immunodermatology, University of Oldenburg, Oldenburg, Germany; ^4^Beamline B23, Diamond Light Source, Didcot, United Kingdom

**Keywords:** gold nanoconjugates, basophils, mast cells, ascomycin, IgE, CD203c, stem cell factor

## Abstract

Calcineurin inhibitors potentially prevent pro-allergic mediator release from basophils and mast cells but are rarely used systemically due to ubiquitous expressions of target signaling proteins. However, specific targeting of allergic effector cells with these inhibitors could circumvent unwanted side effects. We recently demonstrated the biocompatibility of gold nanoparticles (AuNPs) as a platform for non-toxic delivery of signaling inhibitors due to unique physicochemical properties of these nanomaterials. Since AuNPs can be conjugated with both anti-allergic drugs and antibodies or other proteins that specifically recognize basophils and mast cells, our aims were to assess specific targeting of allergic effector cell function using AuNPs conjugated with the calcineurin inhibitor ascomycin. Purified human basophils and LAD2 human mast cells were used for investigations with AuNPs conjugated either to CD203c antibodies or containing stem cell factor (SCF), respectively, which were amine-coupled to acidic groups of reduced glutathione (GSH). GSH was also used as a spacer for immobilization of ascomycin on the gold surface. AuNPs conjugated with anti-CD203c and ascomycin strikingly blocked IgE-dependent degranulation of both purified basophils and those present in mixed leukocyte preparations, suggesting specific targeting of these cells. In contrast, LAD2 mast cell responses were not inhibited using anti-CD203c-containing nanoconjugates but were when the conjugates contained SCF. Successful targeting of allergic effector cells using gold nanoconjugates indicates that this technology may have therapeutic potential for the treatment of allergies by specifically delivering highly effective signaling inhibitors with reduced side effects.

## Introduction

Inflammatory mediator release, (e.g., histamine) from basophils and mast cells plays a major role in contributing to the symptoms of allergic reactions and these cells may also support the underlying tendency for an individual’s immune system to respond in a pro-allergic manner (reviewed in Varricchi et al., 2018). In terms of anti-allergic therapy, it is therefore desirable to target these cells and limit their ability to release inflammatory and immunomodulatory mediators. Several pharmacological inhibitors of intracellular signaling, (e.g., Syk and calcineurin inhibitors) have been shown to substantially reduce the ability of mast cells and basophils to release allergic mediators following stimulation of the high-affinity IgE receptor (Fc𝜀RI) (Oliver et al., 1994; Zuberbier et al., 2001; Plath et al., 2003; Matsubara et al., 2006; Rossi et al., 2006; Harrison et al., 2007). However, due to the ubiquitous expressions of intracellular signals systemic clinical use of these inhibitors is problematical in terms of potential side effects and adverse drug reactions. It is therefore desirable to be able to specifically target effector cells involved in human allergy with drugs that are known to prevent allergen-induced cell activation and the release of pro-inflammatory mediators.

We recently reported the proof-of-principle that gold nanoparticles (AuNPs) could be used to specifically target basophils and other cell types with certain signal transduction inhibitors (Gibbs et al., 2014; Yasinska et al., 2018). Furthermore, compared to most other nanomaterials, AuNPs are relatively non-toxic (Deng et al., 2011; Pissuwan et al., 2011) and are easily conjugated with pharmacological agents and antibodies/antigen-binding peptides (Ma et al., 2010; Gibbs et al., 2014; Yasinska et al., 2018). Moreover, we observed that AuNPs display inherent anti-inflammatory properties themselves by neutralizing the effects of IL-1β (Sumbayev et al., 2013), a cytokine that contributes to allergic inflammation, particularly in asthma.

As a specific marker for mast cells and basophils, we chose CD203c [the ectonucleotide pyrophosphatase/phosphodiesterase family member 3 (ENPP-3)] since it is currently the only known membrane-associated protein that is almost exclusively expressed on both human mast cells and basophils (Bühring et al., 2001; Ghannadan et al., 2002). This marker has been employed in flow cytometric assays for clinical diagnostics regarding basophil activation (Boumiza et al., 2003; Kleine-Tebbe et al., 2006). Its biological function in these cells is not clear but its surface expression is upregulated by priming factors involved in allergic diseases, such as IL-3, and during allergic responses of these cells (Hauswirth et al., 2007; Ono et al., 2010).

Given the above statements, our main aim was to specifically target and inhibit the function of primary human basophils and LAD2 mast cells using AuNPs conjugated to anti-CD203c and ascomycin. Furthermore, we characterized our AuNP-based nanoconjugates (NCJ) using synchrotron radiation circular dichroism (SRCD) spectroscopy. We also compared the effects of NCJ containing anti-CD203c to those conjugated to stem cell factor (SCF). This may allow mast cell-selective targeting since SCF binds to KIT, a receptor that is highly expressed in mature mast cells (other cells include immature haemopoietic stem cells, melanocytes and interstitial cells of the Cajal).

## Methods

### Generation and Characterization of NCJ

Synthesis of 5 nm AuNPs was performed essentially as described previously (Sumbayev et al., 2013; Gibbs et al., 2014; Yasinska et al., 2018). Briefly, 5 ml of aqueous gold (III) chloride trihydrate (10 mM) and 2.5 ml of aqueous sodium citrate (100 mM) were added to 95 ml of milliQ-water in a round bottom flask equipped with a magnetic stirrer. The solution (which has a pale yellow appearance) was cooled down to 1–2°C. Under vigorous stirring 1 ml of aqueous sodium borohydride (4°C, 0.1 M) was added and the solution (which should have a dark red appearance) was then stirred for further 10 min on an ice bath before being allowed to warm up to room temperature. NCJ were generated as described before using glutathione as a linker to immobilize one molecule of anti-CD203c antibody or SCF per 1 nanoparticle. The rest of the surface was covered by glutathiolated (through –COOH group) ascomycin (maximum 1,000 molecules per nanoparticle) (Gibbs et al., 2014; Yasinska et al., 2018). The same principle was adopted for the generation of SCF-containing NCJ (where SCF was employed instead of anti-CD203c). Nanomaterials were characterized using SRCD spectroscopy as described previously (Yasinska et al., 2018).

Briefly, SRCD measurements were conducted using 10 cm path length cell with 3 mm aperture diameter and capacity of 800 μl using the Module B with a 1 nm increment, 1s integration time and 1.2 nm bandwidth at 23°C (Hussain et al., 2012; Yasinska et al., 2018). The results obtained were analyzed using CDAPPs program (Hussain et al., 2012, 2015) and Origin software (OriginLab^TM^).

### Basophil Isolation and Purification

Basophils, were isolated from buffy coat blood (purchased from the NHS Blood and Transplant service following ethics approval (NHS REC 12/WM/0319) by Ficoll-density centrifugation and purified further (to >90%) by immunomagnetic cell sorting using commercial kits, as previously described (Gibbs et al., 2008). Basophil purities were determined by alcian blue staining.

### LAD2 Mast Cells

LAD2 cells were generously provided to us by Dr. A. Kirshenbaum and Prof. D. Metcalfe (NIH, Bethesda, United States) and cultured in Stem-Pro-34 serum-free media in the presence of 100 ng/ml SCF as previously reported (Kirshenbaum et al., 2003). Human SCF protein was produced in *Escherichia Coli* and purified following established protocols (Wang et al., 2008). Cells were sensitized with 100 ng/ml polyclonal IgE (Amsbio, Abingdon, United Kingdom) 24 h before the experiments.

### Cell Stimulation and Histamine Release Assay

Cells were re-suspended in HEPES-buffered Tyrode’s solution (containing 1 mM CaCl_2_) and pre-incubated with or without either NCJ or ascomycin alone (5 or 100 nM) for 15 min at 37° before stimulation (either anti-IgE (1 μg/ml), fMLP (100 nM) or buffer alone) for 30 min. Following centrifugation, histamine content were determined in the supernatants and lysed cell pellets by spectrofluorometric analysis based on the method described by Shore et al. (1959). Histamine releases were calculated by dividing histamine content in respective supernatants by that present in equivalent cell lysates × 100%. Net histamine releases were then calculated by subtracting spontaneous secretions and the results then presented as percentage inhibitions of net histamine release caused by the stimulus alone.

### Statistical Analysis

Each experiment was performed at least three times. When comparing two events at a time we used a two-tailed Student’s *t*-test. Multiple comparisons were performed by ANOVA test and *post hoc* Bonferroni correction was applied. Statistical probabilities (p) were shown in the figures as ^∗^ for *p* < 0.05; ^∗∗^ for *p* < 0.01 and ^∗∗∗^ for *p* < 0.001.

## Results

**FIGURE 1:**
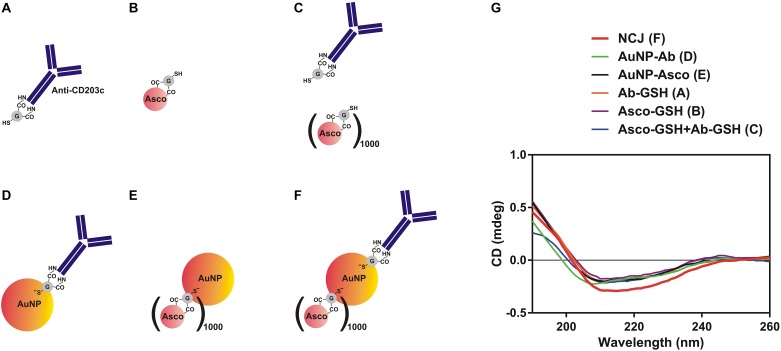
Characterization of nanoconjugates using synchrotron radiation circular dichroism (SRCD) spectroscopy. **(A–F)** Nanomaterials and compounds which were analyzed using SRCD spectroscopy. **(G)** Observed far-UV spectra of anti-CD203c antibody, ascomycin and all possible types of functionalised gold nanoparticles. Data are the mean values of four independent experiments.

Our first objective was to characterize the NCJs using far-UV CD spectra of the components, the materials and compounds comprising the anti-CD203c- and ascomycin-conjugated AuNPs (Figure 1A–F) by use of SRCD spectroscopy (Figure 1G). Our observations confirmed that immobilization of both antibody and the drug was successful.

**FIGURE 2:**
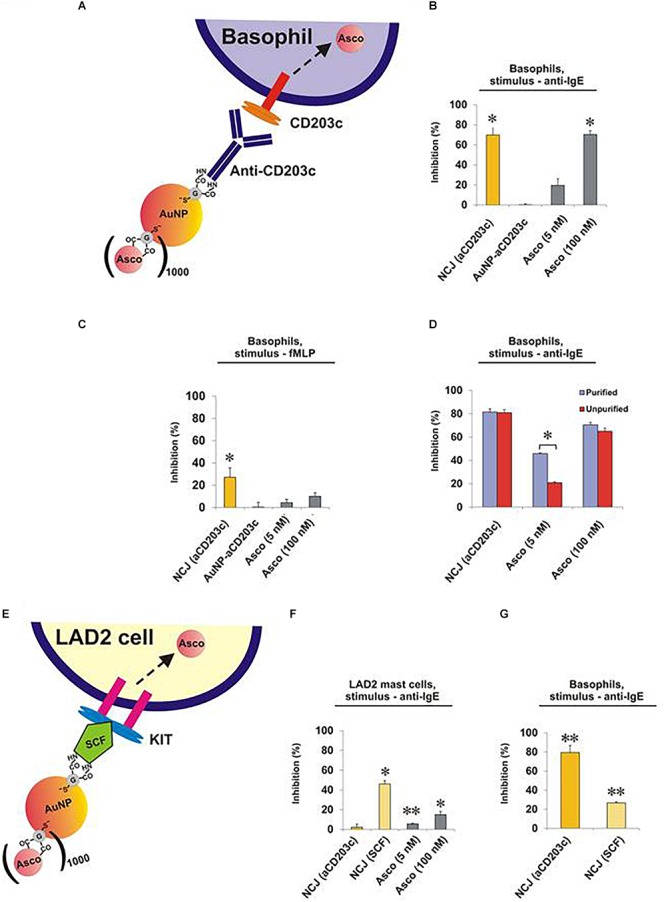
Effect of NCJs on histamine release from human basophils and LAD2 mast cells. Cells were preincubated for 15 min either with NCJs, ascomycin or buffer alone before stimulation for 30 min, after which histamine releases were assessed. All results are shown as percentage inhibition of histamine release ± SEM. ^∗^ and ^∗∗^ denote significant differences from control using a paired Student’s *t*-test (*p* < 0.05 or *p* < 0.01, respectively). Panel **A** Scheme illustrating the interaction of a NCJ containing anti-CD203c with a human basophil and subsequent ascomycin delivery. Panel **B** Basophils stimulated with anti-IgE (*n* = 4). Results were first corrected from spontaneous releases (5.4 ± 1.1%) and percentage inhibition calculated from net anti-IgE-induced release in the absence of NCJs or inhibitors (25.3 ± 4.1%). Panel **C** Basophils stimulated with fMLP (*n* = 4). Results were first corrected from spontaneous releases (4.2 ± 1.2%) and percentage inhibition calculated from net fMLP-induced release in the absence of NCJs or inhibitors (31.6 ± 4.0%). Panel **D** Comparison of the inhibitory effects of NCJs and ascomycin alone in purified (>90% pure) and unpurified (<2% pure) human basophils stimulated with anti-IgE. Spontaneous histamine releases (4.6 ± 0.5% for purified basophils, 3.2 ± 0.7% for unpurified basophils) were first subtracted and the results expressed as percentage inhibition of histamine release caused by anti-IgE alone (net releases were 14 ± 2.7% for purified basophils, 14.2 ± 2.5% for unpurified basophils) (*n* = 8). Panel **E** Scheme illustrating interaction of a NCJ containing SCF with LAD2 mast cell and subsequent ascomycin delivery. Panel **F** Effect of NJCs on histamine release from LAD2 cells stimulated with anti-IgE (*n* = 6). Results were first corrected from spontaneous releases (15.7 ± 0.5%) and percentage inhibition calculated from net anti-IgE-induced release in the absence of NCJs or inhibitors (15.9 ± 0.7%). Panel **G** Effect of different NJCs on histamine release from purified human basophils stimulated with anti-IgE. Results were first corrected from spontaneous releases (4.0 ± 0.3%) and percentage inhibition calculated from net anti-IgE-induced release in the absence of NCJs or inhibitors (10.6 ± 1.0%).

Next, we compared the effects of NCJs and ascomycin alone on histamine release from purified human basophils stimulated either with anti-IgE (Figure 2A,B and Supplementary Figures 1A,B) or the N-formylated tripeptide fMLP (Figure 2C and Supplementary Figures 1C,D). In agreement with our previous observations (Gibbs et al., 2014) NCJs containing ascomycin and anti-CD203c substantially inhibited IgE-dependent basophil histamine release and this level of inhibition was similar to that seen with 100 nM ascomycin alone. Our current results also include the effects of NCJs without ascomycin, which did not show any inhibitory properties. In contrast, NCJs were less effective at inhibiting histamine release from basophils induced by fMLP, although the inhibitory effects with NCJs were still significantly greater than those seen with ascomycin alone at the highest concentration (Figure 2C).

We next asked whether the striking inhibitory properties of NCJs regarding the abrogation of IgE-dependent histamine release from purified basophils were matched in unpurified basophil preparations. Indeed, NCJs had similar inhibitory effects in both preps, while ascomycin alone was less effective (especially at the lower, 5 nM, concentration), presumably due to specific basophil targeting by the NCJs and greater distribution of the free drug between basophils and contaminating peripheral blood mononuclear cells (Figure 2D and Supplementary Figure 2).

Surprisingly, NCJs containing anti-CD203c were not effective at inhibiting anti-IgE-induced histamine release from LAD2 mast cells (Figure 2E,F and Supplementary Figure 3). The effects of ascomycin alone were also less effective than previously observed for basophils, possibly owing to requiring longer preincubation periods than for basophils. Non-etheless, anti-CD203c-containing NCJ were even less effective at inhibiting histamine release from LAD2 mast cells than the moderate inhibition seen with ascomycin alone. In contrast, when coupled to SCF, the inhibitory actions of NCJ were restored to levels greater than seen for ascomycin alone. We also observed that SCF-containing NCJs were moderately (though significantly) effective at inhibiting basophil-derived histamine release, though the inhibitory properties were considerably less compared to anti-CD203c-containing NCJs (Figure 2G).

## Discussion

Our SRCD spectroscopy results demonstrate that immobilization of ascomycin and anti-CD203c on the gold surface of AuNPs doesn’t affect the antibody secondary structure, based on unchanged shape of the respective SRCD spectrum curves (Figure 1G; successful immobilizations of targeting agents on AuNPs is shown in Supplementary Figure 4). This explains preserved biochemical activity of the antibody upon its immobilization on the gold surface. This is confirmed by the functional activities of the NCJs in terms of targeting and inhibiting histamine release from allergic effector cells. For basophils, this inhibitory action was clearly due to the presence of ascomycin on the NCJs, since no effects were observed using NCJs without ascomycin conjugation. Furthermore, the inhibitory actions of ascomycin were more pronounced in terms of inhibiting IgE-dependent histamine release compared to fMLP. As a result, ascomycin-containing NCJs were also less effective at inhibiting fMLP-induced basophil histamine release.

The concentration of ascomycin delivered into basophils within the NCJs was maximally 5 nM, based on stoicheometrical calculations on the number of ascomycin molecules fused to each nanoparticle and the number of nanoparticles incubated with basophils (Gibbs et al., 2014). The increased inhibitory effect of these NCJs compared to incubation with 5 nM ascomycin alone supports that the NCJs facilitate specific cellular targeting. Furthermore, we obtained the same results with the NCJs in both pure and unpure basophils preparations, whereas 5 nM ascomycin alone displayed less inhibitory effects on unpure basophils than pure basophils. This suggests that ascomycin, which is a relatively lipophilic molecule, would otherwise disperse into various cell populations *in vivo*, lowering its effective concentration in basophils and increasing the likelihood of side effects (thus emphasizing the need for cell-specific targeting using NCJs).

In terms of the effects of NCJs on LAD2 mast cells, anti-CD203c conjugation surprisingly failed to target these cells, where no significant inhibition of IgE-dependent histamine release was observed in contrast to ascomycin alone. Indeed, we observed that LAD2 cells expressed barely detectable levels of CD203c (Supplementary Figure 5A). This suggests that CD203c may be absent in certain aberrant mast cell disorders although it is be widely expressed both on a variety of human primary mast cells as well as *in vitro* cultured mast cells and other human mast cell lines (Ghannadan et al., 2002; Andersen et al., 2008; Cop et al., 2017). The LAD2 cell line is derived from a patient with mast cell leukemia/sarcoma (Kirshenbaum et al., 2003). However, neoplastic mast cells from systemic mastocytosis patients have, in contrast to our observations with LAD2 cells, been shown to overexpress CD203c (Hauswirth et al., 2008). This would advocate the use of NCJs containing anti-CD203c (or single chain antibodies/peptides against this protein) to target most other human mast cells and highlights the limitations of LAD2 mast cells in these particular studies.

An alternative mast cell-specific marker is CD117 (KIT), which binds SCF. Our results clearly showed, in contrast to anti-CD203c-containing NCJs, that NCJs containing SCF significantly inhibited LAD2 cell histamine release much more than ascomycin alone. This verifies the concept of specific drug delivery into mast cells using NCJs and shows that CD117 could be used to specifically target mast cells in tissues where other KIT-positive cells (present mainly in the bone marrow) are absent. We assumed that basophils could not be targeted with SCF-containing NCJs since, other than during development, they are generally considered to be CD117/KIT negative (Han et al., 2008). Indeed, Western blot analysis of LAD2 and basophil lysates clearly demonstrated high KIT expression in LAD2 mast cells whereas it was undetectable in basophils (Supplementary Figure 5B). However, SCF-containing NCJs still gave rise to a moderate inhibition of histamine release from basophils. This suggests that SCF may non-specifically interact with some of the basophil cell surface-based receptors or have a moderate affinity to some of them. The second scenario is more likely given that SCF is known to stimulate chemotaxis and the survival of peripheral blood basophils (Heinemann et al., 2005).

We conclude that AuNP-based NCJs are highly effective both at targeting human allergic effector cells and substantially blocking their function by delivering anti-allergic agents. However, expensive stability as well as *in vivo* safety testing will need to be conducted in before this technology can be developed further as a new therapeutic approach in a clinical setting.

## Data Availability

The datasets generated for this study are available on request to the corresponding author.

## Author Contributions

BG conceived the work, together with VS, and conducted the experiments on basophils and LAD2 mast cells together with IY. LC provided and characterized the gold nanoparticles. VS, IY, and BG designed the nanoconjugates. RH, GS, IY, and VS characterized the NCJs using SRCD spectroscopy. UR contributed to analysis and interpretation of the data and critical evaluation of the manuscript content. All authors contributed to writing the manuscript.

## Conflict of Interest Statement

The authors declare that the research was conducted in the absence of any commercial or financial relationships that could be construed as a potential conflict of interest.
