# Supplementary material for: Targeting of Basophil and Mast Cell Pro-Allergic Reactivity Using Functionalised Gold Nanoparticles
Source: Front Pharmacol. 2019 Mar 29;10:333. doi: 10.3389/fphar.2019.00333 (PMC6449467; doi:10.3389/fphar.2019.00333)
Supplement: Supplementary file 1 [file Table_1.DOCX]

**Supplementary data**

**Targeting of Basophil and Mast Cell Pro-Allergic Reactivity using Functionalised Gold Nanoparticles.**

Inna M. Yasinska^1*^, Luigi Calzolai^2^, Ulrike Raap^3^, Rohanah Hussain^4^, Giuliano Siligardi^4^, Vadim V. Sumbayev^1^, Bernhard F. Gibbs^1,3*^

^1^Medway School of Pharmacy, Universities of Kent and Greenwich, Chatham Maritime, United Kingdom

^2^European Commission, Joint Research Centre, Ispra, Italy

^3^Division of Experimental Allergy and Immunodermatology, University of Oldenburg, Oldenburg, Germany

^4^Beamline B23, Diamond Light Source, Didcot, UK

^*^Correspondence:

*apl* Prof. Dr. Bernhard F. Gibbs: [bernhard.gibbs@uni-oldenburg.de](mailto:bernhard.gibbs@uni-oldenburg.de)

Dr. Inna M. Yasinska: [I.Yasinska-24@kent.ac.uk](mailto:I.Yasinska-24@kent.ac.uk)

**Suppl. Fig. 1.** Effect of NJCs on histamine release from human basophils either stimulated with anti-IgE (panels A and B) or fMLP (panels D and F). Cells were preincubated for 15 min with either anti-CD203c-coupled NJC, with or without ascomycin (at a theoretical concentration of ≤5 nM), or ascomycin alone together with positive and negative controls (anti-IgE or buffer alone, respectively). Histamine releases were assessed after 30 min stimulation. Results are shown as relative percentage histamine releases ± SEM for 4 independent experiments (A, C) and corrected for spontaneous releases (B, D). ∗ and ∗∗ denote significant differences from control (p<0.05 or 0.01, respectively).

**Suppl. Fig. 2.** Comparison of the effect of either NJC containing ascomycin or ascomycin alone on histamine release from purified (>90% pure) and unpurified (<2% pure) human basophils from the same donors stimulated with anti-IgE. Cells were preincubated for 15 min with either NJC or ascomycin alone before stimulation with anti-IgE. Histamine releases were assessed after 30 min stimulation. Spontaneous histamine releases (4.6 ± 0.5% for purified basophils, 3.2 ± 0.7% for unpurified basophils) were first subtracted and the results are expressed as percentage net histamine release caused by anti-IgE alone ± SEM (n=8). ** and *** denote significant (p<0.01 and p<0.001, respectively) differences to respective anti-IgE controls. a indicates significant differences of effects between purified and unpurified basophil preparations.

**Suppl. Fig. 3.** Effect of NJCs on histamine release from LAD2 cells stimulated with anti-IgE. Cells were preincubated for 15 min with NJC, containing ascomycin but either conjugated with anti-CD203c or SCF, or ascomycin alone, together with positive and negative controls (anti-IgE or buffer alone, respectively). Histamine releases were assessed after 30 min stimulation. Results are shown as relative percentage histamine releases ± SEM for 4 independent experiments (A) and corrected for spontaneous releases (B). ∗ and ∗∗ denote significant differences (p<0.05 or 0.01, respectively).

Suppl. Fig. 4


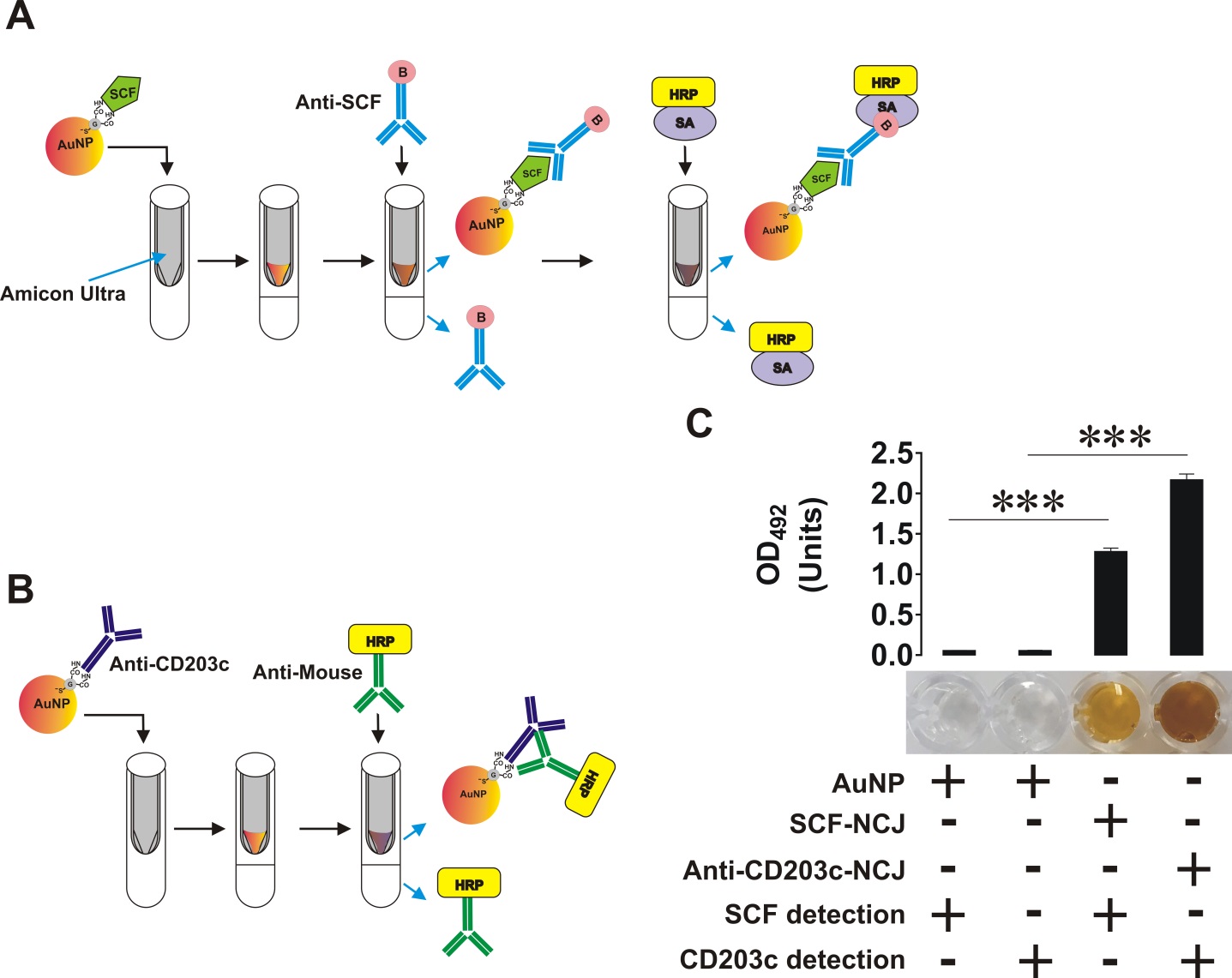


**Suppl. Fig. 4. SCF and anti-CD203c are successfully immobilised on the surface of 5 nm AuNPs.** Nanoconjugates were subjected to a modified ELISA assay where they were exposed to biotinylated antibody against SCF **(A)** or horseradish peroxidase (HRP)-labelled rabbit anti-mouse secondary antibody (recognising anti-CD203c, **(B)**). After each step nanomaterials were collected by centrifugation using Amicon Ultra filters and non-bound proteins were washed away by sterilised bidistilled water. SCF-carrying AuNPs were further exposed to HRP-labelled streptavidin **(A)** followed by the same procedure. Following collection (centrifugation using Amicon Ultra filters) and washing conjugates were exposed to OPD * H_2_O_2_ solution, incubated for 2 min at room temperature followed by 1 min centrifugation in order to separate the solution, which was then straight away supplemented with 10 % H_2_SO_4_ and transferred to the wells of a 96-well plate. The image of the plate was taken and then OD was measured at 492 nm **(C)**. Image is from one experiment representative of four which gave similar results. Data are the mean values ± SEM of four (n =4) independent experiments. *** - p < 0.001 *vs* respective control (as analysed by 2-tailed Student’s t-test).

Suppl. Fig. 5


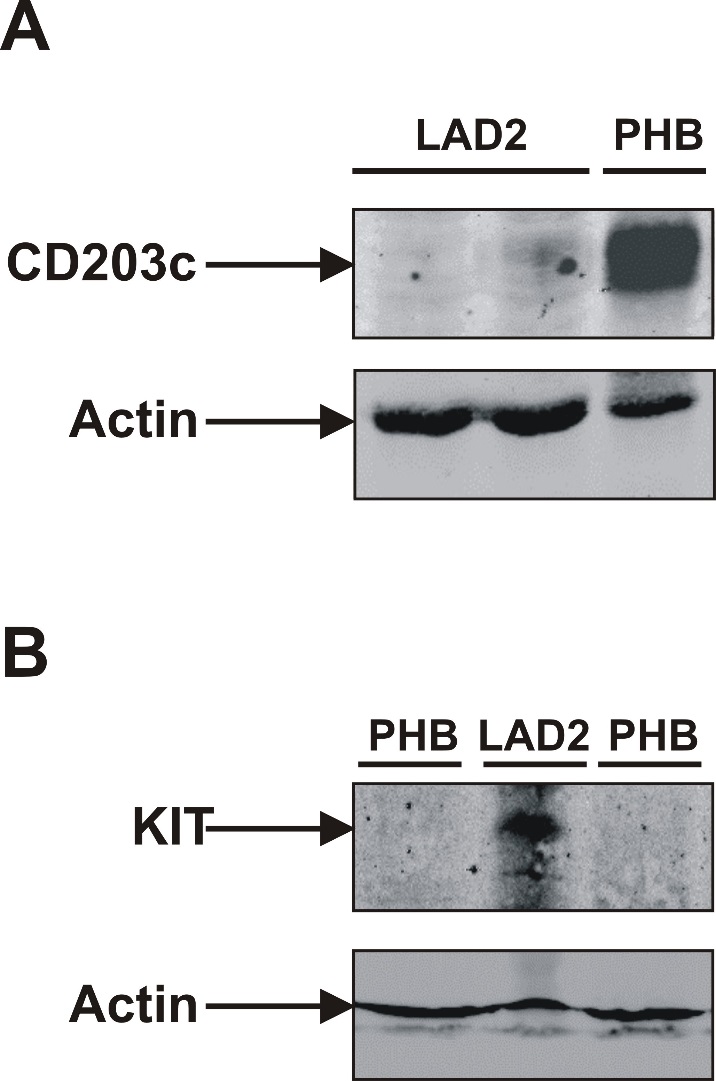


**Suppl. Fig. 5.** **Expression of CD203c and KIT proteins in LAD2 mast cells and primary human basophils.** Cell-associated levels of CD203c **(A)** and KIT **(B)** were analysed in primary human basophils isolated from buffy coats and in LAD2 mast cells by Western blot. Images are from one experiment representative of three which gave similar results. When the result is negative, two batches are shown.
